# Changes in Plasma Short-Chain Fatty Acid Levels after Dietary Weight Loss among Overweight and Obese Adults over 50 Weeks

**DOI:** 10.3390/nu12020452

**Published:** 2020-02-11

**Authors:** Solomon A. Sowah, Frank Hirche, Alessio Milanese, Theron S. Johnson, Mirja Grafetstätter, Ruth Schübel, Romy Kirsten, Cornelia M. Ulrich, Rudolf Kaaks, Georg Zeller, Tilman Kühn, Gabriele I. Stangl

**Affiliations:** 1 German Cancer Research Center (DKFZ), Division of Cancer Epidemiology, Im Neuenheimer Feld 581, 69120 Heidelberg, Germany; t.johnson@Dkfz-Heidelberg.de (T.S.J.); m.grafetstaetter@dkfz-heidelberg.de (M.G.); ruth.schuebel@gmx.de (R.S.); r.kaaks@Dkfz-Heidelberg.de (R.K.); t.kuehn@dkfz-heidelberg.de (T.K.); 2 Medical Faculty, Heidelberg University, 69120 Heidelberg, Germany; 3 Institute of Agricultural and Nutritional Sciences, Martin Luther University Halle-Wittenberg, 06120 Halle (Saale), Germany; frank.hirche@landw.uni-halle.de (F.H.); gabriele.stangl@landw.uni-halle.de (G.I.S.); 4 European Molecular Biology Laboratory (EMBL), Structural and Computational Biology Unit, 69117 Heidelberg, Germany; alessio.milanese@embl.de (A.M.); zeller@embl.de (G.Z.); 5 Biobank of the National Center for Tumor Diseases (NCT) Heidelberg, 69120 Heidelberg, Germany; romy.kirsten@nct-heidelberg.de; 6 Huntsman Cancer Institute and Department of Population Health Sciences, University of Utah, Salt Lake City, UT 84112-5550, USA; neli@hci.utah.edu

**Keywords:** weight loss, short-chain fatty acids, calorie restriction, overweight, obese, adults

## Abstract

Gut microbial-derived short-chain fatty acids (SCFAs) may regulate energy homeostasis and exert anti-carcinogenic, immunomodulatory and anti-inflammatory effects. Smaller trials indicate that dietary weight loss may lead to decreased SCFA production, but findings have been inconclusive. SCFA concentrations were measured by HPLC-MS/MS in plasma samples of 150 overweight or obese adults in a trial initially designed to evaluate the metabolic effects of intermittent (ICR) versus continuous (CCR) calorie restriction (NCT02449148). For the present post hoc analyses, participants were classified by quartiles of weight loss, irrespective of the dietary intervention. Linear mixed models were used to analyze weight-loss-induced changes in SCFA concentrations after 12, 24 and 50 weeks. There were no differential changes in SCFA levels across the initial study arms (ICR versus CCR versus control) after 12 weeks, but acetate concentrations significantly decreased with overall weight loss (mean log-relative change of −0.7 ± 1.8 in the lowest quartile versus. −7.6 ± 2 in the highest, *p* = 0.026). Concentrations of propionate, butyrate and other SCFAs did not change throughout the study. Our results show that weight-loss, achieved through calorie restriction, may lead to smaller initial decreases in plasma acetate, while plasma SCFAs generally remain remarkably stable over time.

## 1. Introduction

Short-chain fatty acids (SCFAs), principally acetate, propionate and butyrate, are produced primarily through bacterial fermentation of indigestible carbohydrates in the colon [1]. To a lesser extent, SCFAs are also produced by the host during catabolism of carbohydrates, proteins and fat [2]. Following the identification of orphan G-protein-coupled receptors, GPR41/FFAR3 and GPR43/FFAR2–predominantly expressed in the colonic epithelium, adipose tissue and immune cells as SCFA receptors [3,4], interest in SCFAs as important signaling molecules in health and disease has increased. Mechanistic studies have demonstrated the potential of SCFAs to alleviate insulin resistance by regulating glucose metabolism [5], to suppress colonic tumorigenesis via the inhibition of histone deacetylase and promotion of apoptosis [6,7], as well as in reducing systemic inflammation by suppressing the production of pro-inflammatory cytokines [8].

Despite preliminary evidence on potential health effects of SCFA, their role in obesity in humans is controversial. By inhibiting lipolysis in adipocytes and increasing basal energy expenditure and lipid oxidation, SCFAs may protect against increased weight gain in humans [9]. Although, long-term studies on SCFA supplementation and weight loss are lacking. Moreover, SCFAs may prevent obesity by suppressing appetite via an upregulation of anorexic hormones such as PYY, GLP-1 and leptin, thereby, reducing ad libitum energy intake in humans [10]. In contrast, it has been suggested that SCFAs may promote obesity through increased energy harvest by contributing up to about 10% of host caloric requirements [1], and relatively higher SCFA concentrations in feces have been observed among obese than among lean individuals [11,12]. An increase in potential for harvesting energy from the diet, due to a distinct gut microbiome composition and activity among the obese, may explain these elevated SCFA concentrations [13].

We have recently reported in a systematic review of human dietary or surgical weight-loss intervention trials that SCFA concentrations, mainly in feces, may decrease with weight loss when fiber intake is reduced [14]. Such decreases in SCFAs may not be desirable considering the potential health benefits of SCFA, outlined above. However, it is important to note that previous studies on weight loss and SCFAs were small, and that SCFAs were mostly measured in faeces, which may not be optimal, considering the limited sample standardization. Furthermore, plasma SCFA, rather than fecal SCFA, has been suggested to be associated with metabolic health [15]. Thus, the impact of weight loss on SCFA concentrations in overweight and obese adults remains largely unclear.

In this study, we report on weight loss-induced changes in plasma SCFA concentrations among 150 overweight or obese adults. These study participants were recruited for the HELENA-Trial, a randomized controlled trial initially carried out to evaluate the metabolic effects of intermittent (ICR) versus continuous calorie restriction (CCR) [16]. Briefly, the study did not show differential effects between ICR and CCR on any of the pre-specified endpoints (changes in the expression of selected genes in adipose tissue, anthropometric measures, body composition parameters and routine metabolic blood biomarkers) over one year. For the present post hoc analyses, we assessed changes in SCFA plasma concentrations, measured at four time points (baseline, and after 12, 24 and 50 weeks), comparing quartiles of overall weight loss, irrespective of the method used to achieve it,, accounting for changes in fiber intake during the trial. Our aim was to investigate whether dietary weight loss induces changes in plasma SCFA over time.

## 2. Materials and Methods

### 2.1. Study Design and Participants

For the HELENA Trial, 150 overweight or obese (BMI > 25 and < 40 kg/m^2^) non-smoking, non-diabetic adults (age range: 35–65 years, 50% women) were recruited at the German Cancer Research Center (DKFZ), Heidelberg, Germany in 2015 to investigate the effects of intermittent versus continuous calorie restriction on metabolic health [17]. Each participant was randomly assigned to one of three dietary regimens; ICR (in form of the so-called 5:2 diet, with 75% calorie reduction on two non-consecutive days per week), CCR (20% daily calorie reduction) or a control regimen over a 50 week period. In brief, the trial period consisted of a 12-week controlled intervention phase with dietary counselling, followed by a 12-week maintenance phase and ended with an observational follow-up phase that lasted for 26 weeks [16]. The trial did not show differential effects of ICR versus CCR in any of the metabolic parameters defined as formal endpoints, i.e., the expression of pre-selected genes that may link obesity and chronic disease risk (primary endpoint) [16]. Likewise, ICR and CCR did not have differential effects on secondary endpoints, i.e., anthropometric and body composition parameters, blood pressure and pulse, and routine blood biomarkers of sugar metabolism, lipid metabolism, steroid hormone signaling or inflammation [16]. The results of the trial have been described by Schübel et al. in detail elsewhere [16]. Approval for the trial was granted by the ethics committee of the Heidelberg University Hospital (Heidelberg, Germany) and it was registered at clinicaltrials.gov (NCT02449148) prior to enrollment. All participants gave written informed consent before they entered the study. The HELENA-Trial was carried out in accordance with the Declaration of Helsinki.

### 2.2. Biospecimen Collection and Laboratory Analysis

Fasting blood samples were collected at 4 different time points, at baseline, and after weeks 12, 24 and 50. Samples were aliquoted and immediately stored at −80 °C until further analyses. Measurements of serum biomarkers (adiponectin, CRP, IL-8, IL-6, IFNγ, TNFα, resistin, leptin, insulin, IGF-1) and other blood-based biomarkers triglycerides, LDL cholesterol, HDL cholesterol, total cholesterol, glucose, GGT, ALT, AST were performed as described in detail elsewhere [16]. All samples were collected and analyzed in accordance with standard operating procedures.

### 2.3. SCFA Analysis

Aliquots of EDTA-plasma were shipped on ice to the Institute of Agricultural and Nutritional Sciences, Martin Luther University Halle-Wittenberg, in June 2018 for the analysis of both free and esterified SCFA concentrations. Specifically, the free circulating and esterified forms of acetate, propionate, butyrate as well as isobutyrate, 2-methylbutyrate, valerate, isovalerate and caproate concentrations were determined by means of high-performance liquid chromatography-tandem mass spectrometry (HPLC-MS/MS) in modification to Chan et al. [18] and Zeng and Cao [19].

For the determination of SCFAs 50 µL of plasma were added to 20 µL of ^13^C-propionic and ^13^C-butyric acid (in 10 mM NaOH) in an HPLC vial (11 mm) and mixed gently. 70 µL of 1 M HClO_4_ were added. Immediately an insert (for 8 mm vials) containing 5 µL of 1 M NaOH was putted as “trap” in the vial. The samples were then incubated at 90 °C for 20 h to bring the volatile fatty acids into the trap solution. This procedure enabled the quantification of total, i.e., free and chemically bound SFCAs. Glycine ethyl ester (GE) was used for derivatization (activated by N-(3-Dimethylaminopropyl)-N′-ethylcarbodiimide hydrochloride (EDC)). The trap solution was mixed with 35 µL of 0.53 M phosphate buffer containing GE (140 mM) and deuterated acetic and deuterated valeric acid. The mixture was added to 10 µL EDC (0.5 M in dry methanol) and mixed. After a reaction time of at least 2 h the samples were measured by HPLC-MS/MS. The coefficients of variation (CVs) (%) for acetate, propionate, and butyrate, were 4.4, 10.5, and 10.8, respectively. The CVs (%) for isobutyrate 2-methylbutyrate, valerate, isovalerate, and caproate, respectively were 13.7, 15.3, 8.9, 11.6, and 9.9.

### 2.4. Statistical Analyses

In cross-sectional analyses, Spearman’s correlation coefficients adjusted for age, sex, and total calorie intake (only for nutrient intake) were used to investigate baseline associations between plasma SCFAs and self-reported nutrient intake (carbohydrates, fats, protein, fiber, and dietary SCFA, derived from 7-day dietary records) [16], as well as routine biomarkers of glucose and lipid metabolism, and inflammation. Correlations of individual SCFA, over time, were also assessed by Spearman’s correlation coefficients.

There were no differences in SCFA over time between the initial study arms (ICR versus CCR. versus control, Supplemental Table S1), and we thus defined quartiles of weight loss irrespective of the dietary method used to achieve it as our primary exposure. This exploratory post hoc categorization was motivated by the fact that SCFAs were not nominal endpoints in the HELENA-Trial, and that we were primarily interested in the effects of overall weight loss, rather than individual calorie restriction approaches on SCFA concentrations in the present project.

For the analyses of overall weight loss, participants were grouped into quartiles based on the amount of weight loss after the controlled intervention phase, i.e., from baseline to week 12. The mean log-relative ± SD changes in weight were 0.02 ± 1.41%, −3.18 ± 0.78%, −5.88 ± 0.75%, and −10.65 ± 2.90% across quartiles 1, 2, 3, and 4, respectively. To examine whether weight loss affected plasma SCFA concentrations, mean ± SE log-relative changes in SCFA concentrations at week 12, 24 and 50 were all calculated relative to baseline SCFA concentrations within each quartile. We used linear mixed models, adjusted for age and sex to analyze the differences in mean relative changes in plasma SCFA concentrations over time between weight loss quartiles. Time, weight loss quartiles group, and time-by-quartile were modelled as fixed effects and participant identifier as the random effect. Differences in the relative changes of the plasma SCFA profiles between the groups were considered to be significant if the *p*-value for the time-by-quartile interaction effect produced by the linear mixed model was < 0.05. To investigate the unique contributions of baseline concentrations of the major SCFAs to post-intervention concentrations, squared semi-partial correlations (R^2^) were obtained for each major SCFA in a regression analysis that included age, sex, initial study arm, and weight loss quartiles as further predictors. In a data-driven approach, we generated principal components from measurements of all 8 SCFAs at 4 different time points, i.e., 32 variables by principal component analysis (PCA) in order to assess interrelationships between all the measured SCFAs. No adjustments for multiple comparisons were made in this analysis. All statistical analyses were performed using SAS Version 9.4 (SAS Institute, Cary, NC, USA).

## 3. Results

### 3.1. Participants Characteristics at Baseline

Out of the 150 participants enrolled into the study, a total of 144, 143 and 136 participants respectively completed the 12, 26 and 50 week study periods. Participants had a mean age of 50.2 ± 8 years, a mean BMI of 33.4 ± 3.8 kg/m^2^, and 50% were female. Baseline characteristics of the participants across weight loss quartiles were similar, as shown in Table 1. The concentrations of SCFA except acetate (slightly lower in Q3) were also similar at baseline (Table 1).

**Figure 1:**
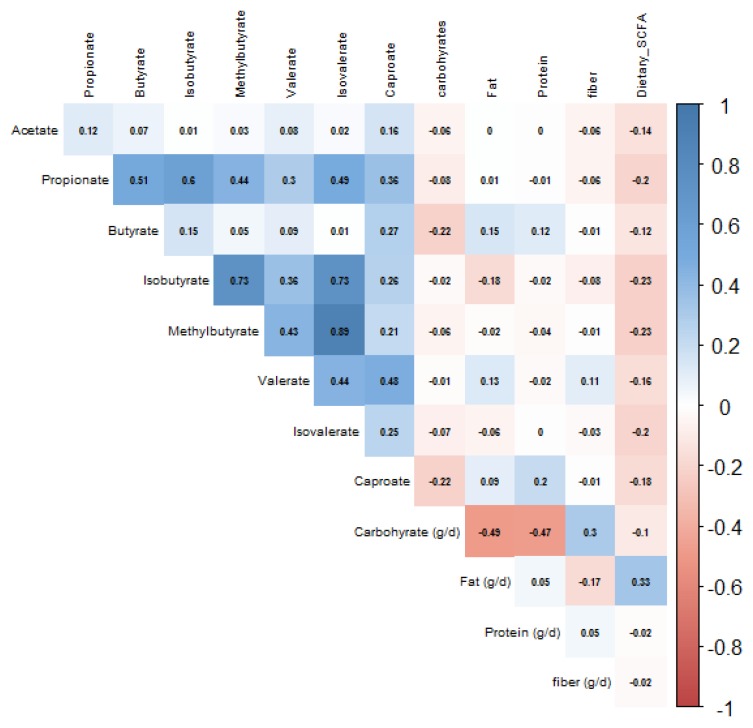
Spearman’s correlation of acetate, propionate, and butyrate with carbohydrate, fat, protein and dietary SCFA intake, *n* = 144.

**Figure 2:**
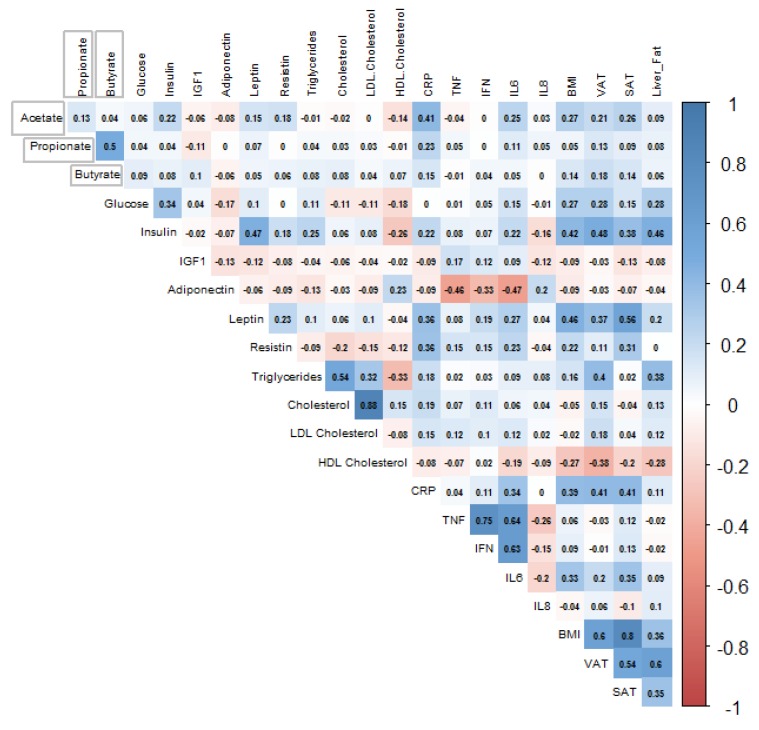
Spearman’s correlation of acetate, propionate, and butyrate with selected routine biomarkers, body composition and percentage liver fat, *n* = 132.

Correlations between plasma SCFA concentrations with self-reported nutrient intake and routine biomarkers are depicted in Figure 1 and Figure 2 respectively. All plasma SCFAs, except for acetate, butyrate and valerate, showed statistically significant (*p* < 0.05), but very weak inverse correlations with dietary SCFA intake. Acetate was positively associated with insulin, resistin, IL-6, BMI, visceral and subcutaneous adipose tissue volumes, although the correlations were very weak (*r* < 0.3). The only slightly stronger correlation was between acetate and CRP levels (*r* = 0.41).

### 3.2. Associations between Weight Loss and SCFA Concentrations

**Figure 3:**
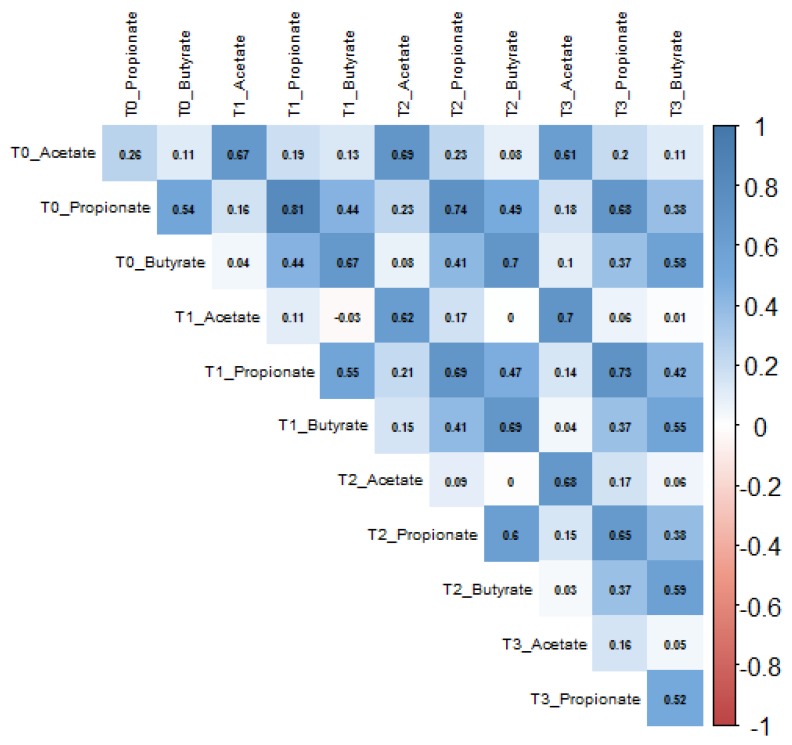
Intra-individual correlations between acetate, propionate, and butyrate concentrations at baseline (T0), week 12 (T1), 24 (T2) and 50 (T3), *n* = 126.

**Figure 4:**
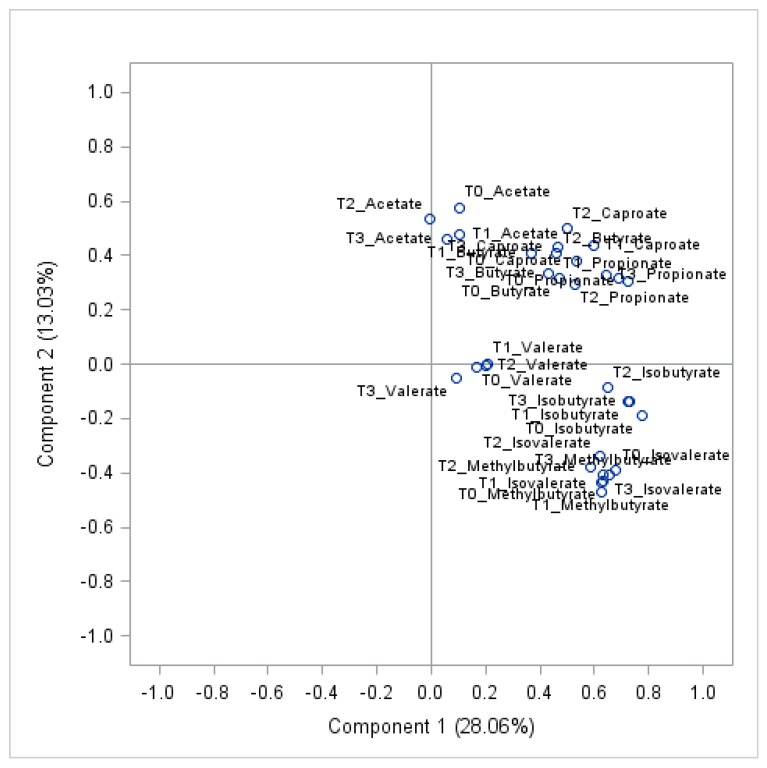
PCA plot of the first two principal components generated from all eight (8) SCFAs at four (4) different time points at baseline (T0), week 12 (T1), 24 (T2) and 50 (T3), *n* = 126.

Concentrations of acetate, butyrate, and propionate showed moderate-to-high positive intra-individual correlations (*r* > 0.5) over time (Figure 3). Similarly, all other SCFAs showed positive intra-individual correlations over time (Supplemental Figure S1). When predicting the variance explained in SCFA concentrations after the 12 weeks intervention by age, sex, weight loss, initial trial arm and baseline SCFA values, the by far greatest part of the variance was explained by baseline SCFA levels, with 30% for acetate, 60% for propionate, and 33% for butyrate, while none of the other predictors explained more than 2%. Further details for acetate, propionate and butyrate at all time points are provided in Supplemental Tables S3–S5. The high intra-individual correlations in SCFA over time are also reflected by the observation that levels of individual SCFA metabolites at all time points showed a strong clustering in PCA (Figure 4).

Apart from acetate, the changes in the concentrations of SCFAs were not significantly different across weight loss quartiles throughout the study (Table 2, Supplemental Figure S2). There was a slight tendency for plasma acetate concentrations to decrease with weight loss (*p*-value of 0.026 for difference between baseline and week 12 across all quartiles). Compared to individuals in the lowest quartile, there was a significantly greater reduction in the concentrations of plasma acetate, 12 weeks after the intervention among those in the highest quartile (−0.7 ± 1.8 mmol/L versus −7.6 ± 2 mmol/L, *p* = 0.015). No statistically significant differences in acetate were observed at week 24 (*p*-value overall = 0.25). While mixed linear models indicated a significant difference in the relative changes in plasma acetate concentrations across weight loss quartiles between baseline and week 50 (*p*-value overall = 0.005), there was no difference in acetate concentrations between the extreme quartiles, i.e., Q1 versus Q4 (−2.4 ± 1.6 mmol/L versus −7.4 ± 2.9 mmol/L, *p* = 0.14).

Dietary records showed that all participants slightly their increased fiber intake (mean ± SD) throughout the study, which may be due to the dietary counselling received during the intervention phase; 17.7 ± 6.9 g/d, 20.6 ± 8.1 g/d and 22.6 ± 20.6 g/d at baseline, week 12, and week 50, respectively (Supplemental Table S6) (no difference across quartiles). However, the reported statistical findings on weight loss and SCFA concentrations did not change upon statistical adjustment for the changes in fiber or carbohydrate intake over time (data not shown).

Characteristics of the study population and intervention effects by initial trial arm (ICR, CCR and Control) are shown in Supplemental Tables S2 and S3. There were no differential effects of ICR versus CCR versus Control on any of the SCFAs.

## 4. Discussion

In this study, we evaluated the effects of moderate dietary weight loss on plasma SCFAs over one year among overweight and obese adults without diabetes. Acetate slightly decreased with weight loss after 12 weeks, but not 24 and 50 weeks, and none of the other SCFAs showed statistically significant changes. Overall, however, the plasma concentrations of SCFAs, particularly those of propionate, remained remarkably stable throughout the study.

With respect to circulating acetate, both positive and inverse associations with obesity have been shown [20,21]. Our finding of a slight initial decrease in acetate with weight loss is in agreement with that reported from a study by Dao et al., in which serum acetate concentrations decreased significantly after 6 and 12 weeks of dietary weight loss among overweight and obese, but otherwise healthy adults [22]. In that study, a decline in the potential for microbial energy harvest after weight loss due to shifts in the composition of the intestinal microbiome could explain the observed decrease in acetate concentrations [13]. Dao et al., showed serum acetate concentration to be positively associated with fecal abundance of the bacterium *Akkermansia muciniphila* at baseline, with a subsequent simultaneous decline in the abundance of this bacterium and serum acetate after 6 weeks of weight loss [22]. Alternatively, reductions in acetate may also be due to a compensatory mechanism, related to the oxidation and utilization of the metabolite as alternative fuel during extended durations of calorie restriction [23,24].

When interpreting our findings on acetate, it should be noted that we measured total acetate, with a large fraction being chemically bound to plasma proteins, unlike previous studies, in which free circulating acetate was measured [15,20]. Acetate is used for acetylation, a common post-translational modification of proteins affecting protein stability, gene expression and protein-protein interactions, which mainly occurs on the lysine residues of proteins [25]. Acetyllysine was detected in more than 20% of mitochondrial proteins [26] and nearly every enzyme involved in glycolysis, gluconeogenesis, citric acid cycle, fatty acid metabolism has been found to be acetylated in the human liver [27]. Thus, it can be assumed that protein acetylation plays an important role in the regulation of energy metabolism, and changes in energy metabolism may affect acetate pools required to regulate protein function. The most abundant circulating protein is albumin, which functions as a transporter of fatty acids, hormones and other metabolites. Several acetylation sites have been characterized in albumin, and also in other plasma proteins, such as fibrinogen [28]. Therefore, the slight reduction in plasma acetate among individuals undergoing weight reduction in our study may well reflect altered post-translational protein modification [27]. However, it should also be noted that the changes in acetate, in our study, were modest and only statistically significant at week 12, but not at later time points. In addition, we cannot explain why acetate levels tended to decrease among study participants in weight loss quartile 2 and 4, but not among those in quartile 3 after 12 weeks. Thus, we cannot rule out that the statistically significant decrease in acetate observed for quartile 4 after 12 weeks was due to chance, unless replicated by further studies.

We did not find evidence for any weight loss effect on propionate and butyrate concentrations in plasma. Apart from rapid gut and hepatic absorption and utilization of SCFAs as energy source, the lack of changes in the concentrations of propionate and butyrate may be related to the fact that participants in this study slightly increased their fiber intake throughout the intervention. By contrast, in previous studies that reported decreased concentrations of SCFAs among participants after weight loss [29,30,31,32], participants consumed extremely low-carbohydrate (<5% of energy) diets, with low amounts of fiber. It is noteworthy, however, that these studies quantified SCFAs in faeces, and cannot be directly compared to our findings on plasma SCFAs.

The production of SCFAs, aside many factors, such as gut bacteria composition and activity, as well as physiological state, is influenced to a large extent by macronutrient intake, especially non-digestible carbohydrate intake [33]. In this context, we were surprised not to observe correlations between questionnaire-derived fiber intake and plasma SCFAs in this study. Strong positive associations between carbohydrates and SCFAs have mostly been shown in studies with very high (above recommended daily allowance) fiber or resistant starch interventions [34,35]. Considering the relatively low intra-individual fluctuations in the major plasma SCFA concentrations over time in our study, our data are not in-line with the notion that SCFA concentrations may be affected by weight loss [14], but rather, they may be under tighter homeostatic control.

To the best of our knowledge, this is the first study to report on the changes in plasma SCFA concentrations, after weight loss in a dietary intervention trial, over one year. A limitation is that we only assessed SCFA changes in peripheral plasma. We did not have the opportunity to investigate their origins (i.e., microbial-derived versus endogenous), although, our findings of moderate to strong intra-individual correlations, in some SCFAs over time, are not in line with the hypothesis that circulating SCFAs are highly variable and affected by the prevailing physiological or homeostatic state [36],. As such the contribution of microbiome-derived acetate to the peripheral concentrations of total, i.e., free and chemically bound SCFAs measured in this study is largely unclear. This limitation could, in part, be circumvented by sampling portal blood for SCFA analysis, but this is not feasible to achieve among healthy adults such as those in our cohort [37,38]. Dietary information used for the analysis was based on self-report, and the possibility of misreporting is acknowledged. Gut-derived hormones, particularly GLP-1, through which circulating SCFA may affect weight control, were not measures in our study [10]. Generally, the present analyses were carried out in a post hoc manner, even though we think our data provides a valid model for investigating the effects of moderate diet-induced weight loss on SCFAs at slightly increasing fiber intake. Finally, sensitivity challenges have previously been reported with the use of liquid chromatography for SCFAs, especially butyrate and propionate analyses in blood [38,39]. The concentrations of acetate reported in this study are higher than those previously reported among adults [40]. As mentioned above, one reason for this difference is that we measured total rather than free acetate. Another reason could be excess hydrolysis of acetyl esters as a result of freezing and thawing or the combination of strong acid and high temperature used in the preparation steps, prior to HPLC-MS/MS analysis [40]. The interpretation of the findings from this study should, therefore, be made in consideration of the aforementioned limitations.

## 5. Conclusions

In the present study, plasma concentrations of free and chemically bound SCFAs remained largely unaffected by moderate diet-induced weight loss among overweight or obese non-diabetics over one year. Overall, fluctuations in plasma SCFAs may be lower than previously thought and future studies comparing the effects of diet and weight loss on SCFAs in different bio-samples, beyond peripheral blood are needed.

## Figures and Tables

**Table 1:** Baseline Characteristics of the Trial Participants across Quartiles of Weight Loss over 12 weeks ^1^.

	Q 1	Q 2	Q 3	Q 4
Mean relative weight loss,%	0.02 ± 1.41	−3.18 ± 0.78	−5.88 ± 0.75	−10.65 ± 2.90
Age, y	51.0 ± 6.3	51.2 ± 8.3	51.2 ± 7.8	47.4 ± 8.3
Weight, kg	94.2 ± 15.8	94.0 ± 14.0	93.3 ± 15.5	95.0 ± 14.2
Height, cm	171.2 ± 10.5	173.8 ± 9.8	173.4 ± 10.7	173.3 ± 7.9
BMI, kg/m^2^	32.1 ± 4.1	31.1 ± 3.7	30.9 ± 3.4	31.5 ± 3.7
Waist circumference, cm	105.6 ± 12.3	105.5 ± 10.7	102.4 ± 11.4	103.9 ± 11.8
Visceral adipose tissue volume, cm^3^	5253.4 ± 2248.9	4963.7 ± 2201.0	4836.2 ± 2006.2	4719.5 ± 2037.5
Subcutaneous adipose tissue volume, cm^3^	13106.8 ± 4620.1	11227.1 ± 2833.4	12117.6 ± 3933.1	12870.9 ± 4046.6
Liver fat,%	7.1 ± 4.4	8.8 ± 7.8	7.9 ± 6.5	7.4 ± 4.9
Systolic blood pressure, mmHg	139.6 ± 11.0	132.2 ± 14.0	136.6 ± 14.4	140.0 ± 21.9
Diastolic blood pressure, mmHg	90.1 ± 8.1	86.0 ± 8.1	87.3 ± 7.7	86.9 ± 9.8
Leptin, ng/mL	29.7 ± 25.1	20.4 ± 19.9	21.2 ± 15.1	29.4 ± 29.0
HOMA-IR	3.4 ± 1.9	2.9 ± 1.8	2.5 ± 1.2	2.6 ± 1.3
Insulin, mU/L	14.7 ± 7.8	12.6 ± 7.4	10.8 ± 5.1	11.2 ± 5.4
Glucose, mg/dL	93.4 ± 7.9	93.2 ± 6.8	94.8 ± 6.8	91.8 ± 8.0
IGF-1, ng/mL	114.6 ± 34.3	124.3 ± 34.1	111.3 ± 33.3	105.1 ± 27.0
HbA1C,%	5.4 ± 0.4	5.5 ± 0.3	5.5 ± 0.3	5.4 ± 0.3
Triglycerides, mg/dL	139.4 ± 64.9	136.1 ± 89.3	143.9 ± 93.2	108.3 ± 53.5
Cholesterol, mg/dL	211.4 ± 34.1	202.1 ± 35.9	214.4 ± 36.0	203.3 ± 34.5
LDL cholesterol^7^, mg/dL	129.5 ± 26.0	120.7 ± 25.0	128.8 ± 26.5	128.7 ± 29.5
HDL cholesterol, mg/dL	54.0 ± 15.0	52.6 ± 14.3	56.8 ± 13.7	52.9 ± 14.9
SCFAs *				
Acetate, mmol/L	5.4 ± 0.6	5.4 ± 0.7	5.0 ± 0.6	5.2 ± 0.6
Propionate, µmol/L	5.7 ± 1.5	6.4 ± 1.9	6.1 ± 1.7	5.5 ± 1.5
Butyrate, µmol/L	0.9 ± 0.3	1.1 ± 0.4	1.0 ± 0.4	0.9 ± 0.3
Isobutyrate, µmol/L	1.9 ± 0.7	2.0 ± 0.6	2.0 ± 0.7	1.9 ± 0.6
2-Methylbutyrate, µmol/L	1.0 ± 0.3	1.2 ± 0.4	1.1 ± 0.4	1.1 ± 0.4
Valerate, µmol/L	0.5 ± 0.4	0.4 ± 0.1	0.5 ± 0.3	0.6 ± 1.1
Isovalerate, µmol/L	3.2 ± 1.1	3.8 ± 1.6	3.4 ± 1.3	3.4 ± 1.1
Caproate, µmol/L	1.6 ± 0.8	1.6 ± 0.7	1.6 ± 0.8	1.4 ± 0.5

^1^*n* = 36 for each quartile; values are presented as mean ± SD; WL–amount of weight loss in kg; SCFA-short chain fatty acid; Q1-quartile 1; Q2-quartile 2; Q3-quartile 3; Q4-quartile 4. * Both free circulating and esterified forms of each SCFA was measured.

**Table 2:** Effects of weight loss on plasma SCFA concentrations between quartiles of weight loss ^1^.

	Mean Log-Relative Changes (Baseline to Week 12)				*p*- Overall ^2^
	Q 1	Q 2	Q 3	Q 4	
Acetate	−0.7 ± 1.8	−5.9 ± 2.2	−0.8 ± 1.5	−7.6 ± 2	0.026
					Q1 vs. Q2 (0.09)
					Q1 vs. Q3 (0.99)
					Q1 vs. Q4 (0.015) *
Propionate	2.5 ± 3	0.8 ± 2.8	−1.6 ± 3.8	8.8 ± 3.6	0.20
Butyrate	8.3 ± 5.2	−0.8 ± 5.6	0.3 ± 3.9	14.8 ± 5.4	0.26
Isobutyrate	−4.8 ± 3.8	−7.7 ± 3.9	−10.9 ± 4.2	3.8 ± 3.7	0.10
2-Methylbutyrate	−2.2 ± 4.5	−11.6 ± 5.8	−1.9 ± 5.3	−0.1 ± 4.8	0.33
Valerate	3.1 ± 4.4	0.9 ± 3.6	6.8 ± 5.8	7.7 ± 4.9	0.83
Isovalerate	−3 ± 5	−1.8 ± 5.9	2.6 ± 5.6	1.6 ± 4.4	0.98
Caproate	5.5 ± 3.7	1.8 ± 4.5	2.3 ± 5.1	5.5 ± 4.7	0.96

^1^*n* = 144; ^2^ *P* overall is the *p*-value of the group (quartile) by time interaction from linear mixed models; Q1-quartile 1; Q2-quartile 2; Q3-quartile 3; Q4-quartile 4; * significant difference between groups.

## Supplementary Materials

The following are available online at https://www.mdpi.com/2072-6643/12/2/452/s1, Figure S1: Correlations between other SCFAs across time points. T0-baseline, T1-week 12, T2-week 24, T3-week 50, Figure S2: (**a**) Changes in acetate according to weight loss quartiles; (**b**) Changes in propionate according to weight loss quartiles; (**c**) Changes in butyrate according to weight loss quartiles, Table S1: Baseline Characteristics, Blood-Based Biomarkers and SCFA Concentrations of Participants according to Treatment Group, Table S2: Effects of Weight Loss on Plasma SCFA Concentrations between Study Arms, Table S3: Summary of Multiple Regression Analysis for Variables Predicting Acetate. Concentrations at week 12, 24 and 50, Table S4: Summary of Multiple Regression Analysis for Variables Predicting Propionate Concentrations at Week 12, 24 and 50, Table S5: Summary of Multiple Regression Analysis for Variables Predicting Butyrate Concentrations at Week 12, 24 and 50, Table S6: Reported Energy and Macronutrient Intake at Baseline, and Reported Relative Changes Across Quartiles over Time at Week 12 and 50.
