# Supplementary material for: Changes in Plasma Short-Chain Fatty Acid Levels after Dietary Weight Loss among Overweight and Obese Adults over 50 Weeks
Source: Nutrients. 2020 Feb 11;12(2):452. doi: 10.3390/nu12020452 (PMC7071291; doi:10.3390/nu12020452)
Supplement: Supplementary file 1 [file nutrients-12-00452-s001.pdf]

Electronic Supplementary Material

Changes in Plasma Short-Chain Fatty Acid Levels after Dietary Weight Loss among Overweight and Obese Adults over 50 Weeks

Solomon A. Sowah [1,2,*], Frank Hirche [3], Alessio Milanese [4], Theron S. Johnson [1], Mirja Grafetstätter [1,2], Ruth Schübel [1], Romy Kirsten [5], Cornelia M. Ulrich [6], Rudolf Kaaks [1], Georg Zeller [4], Tilman Kühn [1] and Gabriele I. Stangl [3]

[1] German Cancer Research Center (DKFZ), Division of Cancer Epidemiology, Im Neuenheimer Feld 581, 69120 Heidelberg, Germany; s.sowah@dkfz.de
[2] Medical Faculty, Heidelberg University, Heidelberg, Germany
[3] Institute of Agricultural and Nutritional Sciences, Martin Luther University Halle-Wittenberg, 06120, Halle (Saale), Germany
[4] European Molecular Biology Laboratory (EMBL), Structural and Computational Biology Unit, 69117 Heidelberg, Germany
[5] Biobank of the National Center for Tumor Diseases (NCT) Heidelberg, Heidelberg, Germany
[6] Huntsman Cancer Institute and Department of Population Health Sciences, University of Utah, Salt Lake City, UT 84112-5550, USA
* Correspondence: s.sowah@dkfz.de

Received: 14 January 2020; Accepted: 06 February 2020; Published: date

Supplemental Table S1. Baseline Characteristics, Blood-Based Biomarkers and SCFA Concentrations of Participants according to Treatment Group [1].

	ICR (*n* = 49, *w* = 49%)	CCR (*n* = 49, *w* = 49%)	Control (*n* = 52, *w* = 52%)
Age, y	49.4 ± 9.0	50.5 ± 8.0	50.6 ± 7.1
Weight, kg	96.4 ± 15.8	92.5 ± 15.7	93.3 ± 13.3
Height, cm	173.3 ± 9.7	171.9 ± 9.9	173.1 ± 9.9
BMI, kg/m^2	32.0 ± 3.7	31.2 ± 4.0	31.1 ± 3.6
Waist circumference, cm	104.7 ± 12.3	103.7 ± 11.9	104.2 ± 10.7
Hip circumference, cm	110.5 ± 9.8	108.4 ± 10.2	109.7 ± 9.4
Visceral adipose tissue volume, cm^3	4817.8 ± 1889.1	4894.5 ± 2178.3	4943.1 ± 2267.4
Subcutaneous adipose tissue volume, cm^3	12821.6 ± 4267.2	12193.1 ± 3996.6	11944.8 ± 3845.2
Liver fat,%	7.7 ± 4.6	8.3 ± 8.0	7.1 ± 4.7
Systolic blood pressure, mmHg	139.4 ± 18.7	136.0 ± 16.7	136.0 ± 12.5
Diastolic blood pressure, mmHg	87.2 ± 9.9	87.3 ± 8.7	87.8 ± 7.3
Leptin, ng/mL	25.2 ± 24.0	23.3 ± 19.8	25.9 ± 25.0
HOMAIR	2.7 ± 1.3	3.0 ± 1.7	3.0 ± 1.8
Insulin, mU/L	11.6 ± 5.4	12.6 ± 6.9	12.7 ± 7.3
Glucose, mg/dL	92.7 ± 7.5	93.9 ± 7.5	93.5 ± 7.4
IGF-1, ng/mL	108.9 ± 31.3	115.1 ± 36.6	121.4 ± 32.1
HbA1C,%	5.4 ± 0.3	5.5 ± 0.4	5.5 ± 0.2
Triglycerides, mg/dL	130.0 ± 83.8	121.2 ± 66.3	145.0 ± 85.5
Cholesterol, mg/dL	205.0 ± 30.8	202.9 ± 39.3	211.8 ± 36.1
LDL cholesterol, mg/dL	124.5 ± 22.4	122.5 ± 31.5	130.4 ± 27.3
HDL cholesterol, mg/dL	54.1 ± 14.3	56.2 ± 16.3	51.8 ± 11.8
**SCFAs** *			
Acetate, mmol/L	5.2 ± 0.5	5.3 ± 0.7	5.3 ± 0.7
Propionate, µmol/L	5.8 ± 1.9	5.9 ± 1.6	6.0 ± 1.5
Butyrate, µmol/L	0.9 ± 0.3	0.9 ± 0.4	1.0 ± 0.4
Isobutyrate, µmol/L	1.9 ± 0.7	2.0 ± 0.5	1.9 ± 0.6
2-Methylbutyrate, µmol/L	1.1 ± 0.5	1.1 ± 0.3	1.1 ± 0.4
Valerate, µmol/L	0.6 ± 1.0	0.4 ± 0.1	0.5 ± 0.3
Isovalerate, µmol/L	3.6 ± 1.4	3.4 ± 1.1	3.3 ± 1.3
Caproate, µmol/L	1.5 ± 0.6	1.4 ± 0.5	1.6 ± 0.9

[1] *n* = 150. Values are presented as means ± SD. ICR-intermittent calorie restriction, CCR-continuous calorie restriction; Groups were comparable at baseline in all parameters. * Both free circulating and esterified forms of each SCFA were measured.

Supplemental Table S2. Effects of Weight Loss on Plasma SCFA Concentrations between Study Arms [1].

		Baseline	Log_e Relative Change [2] (wk 12)	Log_e Relative Change [2] (wk 24)	Log_e Relative Change [2] (wk 50)
Acetate	ICR	5.2 ± 0.5	−3.3 ± 1.4	−1.7 ± 1.6	−4.4 ± 1.6
	CCR	5.3 ± 0.7	−4.2 ± 1.6	1.8 ± 2.2	−3.6 ± 2.3
	Control	5.3 ± 0.7	−3.7 ± 2.0	−2.6 ± 1.8	−2.1 ± 1.8
Propionate	ICR	5.8 ± 1.9	7.6 ± 3.5	9.9 ± 3.8	1.7 ± 4.2
	CCR	5.9 ± 1.6	2.4 ± 2.7	5.1 ± 4.4	1.7 ± 4.2
	Control	6.0 ± 1.5	−1.6 ± 2.5	6.3 ± 3.5	1.7 ± 3.6
Butyrate	ICR	0.9 ± 0.3	12.7 ± 5.1	22.9 ± 3.6	0.9 ± 5.9
	CCR	1.0 ± 0.4	7.2 ± 4.2	11.2 ± 5.7	−0.3 ± 5.5
	Control	1.0 ± 0.4	−1.9 ± 3.9	6.0 ± 4.4	−4.1 ± 4.2
Isobutyrate	ICR	2.0 ± 0.8	−1.2 ± 4.1	5.6 ± 4.6	1.0 ± 2.9
	CCR	2.0 ± 0.5	−12.2 ± 3.2	−8.1 ± 4.6	−3.9 ± 4.0
	Control	1.9 ± 0.6	−1.8 ± 2.9	6.1 ± 3.6	4.6 ± 3.6
2-Methylbutyrate	ICR	1.1 ± 0.5	−3.2 ± 5.1	4.1 ± 6.1	−0.5 ± 4.4 *
	CCR	1.1 ± 0.3	−11.0 ± 3.8	−3.6 ± 4.1	0.1 ± 4.4
	Control	1.1 ± 0.4	1.6 ± 4.3	6.3 ± 4.7	12.7 ± 4.8
Valerate	ICR	0.6 ± 1.0	3.4 ± 3.5	11.6 ± 4.3	3.1 ± 4.7
	CCR	0.4 ± 0.1	9.2 ± 4.3	15.0 ± 4.9	18.9 ± 9.5
	Control	0.5 ± 0.3	1.7 ± 4.3	5.9 ± 5.6	5.1 ± 5.6
Isovalerate	ICR	3.6 ± 1.4	2.2 ± 5.5	0.7 ± 5.8	−0.6 ± 4.6
	CCR	3.4 ± 1.1	−3.0 ± 3.5	−2.3 ± 5.0	2.9 ± 4.9
	Control	3.4 ± 1.3	0.2 ± 4.4	3.0 ± 4.1	11.0 ± 5.1
Caproate	ICR	1.5 ± 0.6	2.2 ± 4.2	11.6 ± 4.0	4.1 ± 4.0
	CCR	1.4 ± 0.5	7.4 ± 4.2	17.8 ± 4.1	15.9 ± 5.6
	Control	1.6 ± 0.9	1.9 ± 3.4	5.0 ± 4.3	3.1 ± 5.6

[1] n = 144. Both free circulating and esterified forms of each SCFA were measured. Baseline values are means±SD. [2] Means (±SEM) of individual log_e relative changes between baseline and post-intervention. SCFA-short-chain fatty acid; ICR-intermittent calorie restriction; CCR-continuous calorie restriction. * Significant difference between treatment groups.

Supplemental Table S3. Summary of Multiple Regression Analysis for Variables Predicting Acetate. Concentrations at week 12, 24 and 50 [1.]

Predictors	β	SE	*p*-Value	R^2
**Dependent Variable: T1_Acetate, *n* = 138**				
Intercept	2.26	0.53	0.0001	.
T0_Acetate	0.59	0.08	0.0001	0.26
Age, y	−0.01	0.01	0.0887	0.01
sex 1	−0.10	0.10	0.3116	0.00
sex 2	0.00	.	.	.
Q1	0.51	0.14	0.0004	0.06
Q2	0.22	0.14	0.1071	0.01
Q3	0.33	0.14	0.0166	0.03
Q4	0.00	.	.	.
ICR	0.12	0.12	0.3295	0.00
CCR	0.02	0.11	0.8933	0.00
Control	0.00	.	.	.
**Dependent Variable: T2_Acetate, *n* = 131**				
**Predictors**	**β**	**SE**	***p*-value**	R^2
Intercept	0.83	0.62	0.1867	.
T0_Acetate	0.82	0.09	0.0001	0.35
Age, y	0.00	0.01	0.7966	0.00
sex 1	−0.08	0.12	0.4739	0.00
sex 2	0.00	.	.	.
Q1	0.23	0.17	0.1783	0.01
Q2	0.13	0.17	0.4563	0.00
Q3	0.26	0.16	0.1077	0.01
Q4	0.00	.	.	.
ICR	0.05	0.14	0.7097	0.00
CCR	0.19	0.14	0.1715	0.01
Control	0.00	.	.	.
**Dependent variable: T3_Acetate, *n* = 130**				
**Predictors**	**β**	**SE**	***p*-Value**	R^2
Intercept	1.53	0.56	0.0075	.
T0_Acetate	0.62	0.08	0.0001	0.26
Age, y	0.01	0.01	0.3935	0.00
sex 1	−0.15	0.10	0.1596	0.01
sex 2	0.00	.	.	.
Q1	0.27	0.15	0.0870	0.02
Q2	0.04	0.15	0.7967	0.00
Q3	0.35	0.15	0.0186	0.03
Q4	0.00	.	.	.
ICR	−0.10	0.13	0.4199	0.00
CCR	−0.08	0.13	0.5072	0.00
Control	0.00	.	.	.

[1] T0_Acetate, T1_Acetate, T2_Acetate, and T3_Acetate–acetate concentrations at baseline, wk 12, wk 24 and wk 50 respectively. Q1, Q2, Q3 and Q4–weight loss quartiles 1, 2, 3, and 4 respectively; ICR, CCR and

Control–intermittent calorie restriction, continuous calorie restriction, and control groups respectively of the initial study arms; sex 1-male, sex 2-female. R^2–squared semi-partial correlation coefficient.

Supplemental Table S4. Summary of Multiple Regression Analysis for Variables Predicting Propionate Concentrations at Week 12, 24 and 50 [1].

**Dependent Variable: T1_Propionate, *n* = 138**				
**Predictors**	**β**	**SE**	***p*-Value**	***R*²**
Intercept	1.66	0.72	0.0235	.
T0_Propionate	0.78	0.06	0.0001	0.58
Age, y	−0.01	0.01	0.6461	0.00
sex 1	0.07	0.19	0.7020	0.00
sex 2	0.00	.	.	.
Q1	−0.17	0.28	0.5298	0.00
Q2	−0.12	0.28	0.6600	0.00
Q3	−0.34	0.27	0.2106	0.00
Q4	0.00	.	.	.
ICR	0.38	0.24	0.1130	0.01
CCR	0.25	0.23	0.2719	0.00
Control	0.00	.	.	.
**Dependent Variable: T2_Propionate, *n* = 131**				
**Predictors**	**β**	**SE**	***p*-Value**	***R*²**
Intercept	2.51	1.25	0.0476	.
T0_Propionate	0.81	0.10	0.0001	0.36
Age, y	0.00	0.02	0.9606	0.00
sex 1	−0.65	0.32	0.0432	0.02
sex 2	0.00	.	.	.
Q1	−0.47	0.48	0.3357	0.00
Q2	−0.42	0.49	0.3915	0.00
Q3	−0.62	0.47	0.1888	0.01
Q4	0.00	.	.	.
ICR	−0.21	0.41	0.6111	0.00
CCR	−0.08	0.40	0.8456	0.00
Control	0.00	.	.	.
**Dependent Variable: T3_Propionate, *n* = 130**				
**Predictors**	**β**	**SE**	***p*-Value**	***R*²**
Intercept	2.35	0.87	0.0075	.
T0_Propionate	0.55	0.06	0.0001	0.36
Age, y	0.02	0.01	0.3008	0.01
sex 1	−0.33	0.22	0.1299	0.01
sex 2	0.00	.	.	.
Q1	−0.19	0.33	0.5686	0.00
Q2	−0.09	0.33	0.7912	0.00
Q3	−0.38	0.32	0.2331	0.01
Q4	0.00	.	.	.
ICR	-0.21	0.27	0.4410	0.00
CCR	0.08	0.27	0.7819	0.00
Control	0.00	.	.	.

[1] T0_Propionate, T1_ Propionate, T2_ Propionate, and T3_ Propionate–propionate concentrations at baseline, wk 12, wk 24 and wk 50 respectively. Q1, Q2, Q3 and Q4–weight loss quartiles 1, 2, 3, and 4 respectively; ICR, CCR and Control–intermittent calorie restriction, continuous calorie restriction, and control groups respectively of the initial study arms; sex 1-male, sex 2-female. R^2–squared semipartial correlation coefficient.

Supplemental **Table S5.** Summary of Multiple Regression Analysis for Variables Predicting Butyrate Concentrations at Week 12, 24 and 50 [1].

**Dependent Variable: T1_Butyrate, *n* = 138**				
**Predictors**	**β**	**SE**	***p*-Value**	R^2
Intercept	0.48	0.18	0.0099	.
T0_Butyrate	0.59	0.07	0.0001	0.33
Age, y	0.00	0.00	0.6062	0.00
sex 1	0.08	0.05	0.1078	0.01
sex 2	0.00	.	.	.
Q1	−0.01	0.07	0.9105	0.00
Q2	−0.02	0.08	0.7467	0.00
Q3	−0.07	0.07	0.3530	0.00
Q4	0.00	.	.	.
ICR	0.04	0.06	0.4937	0.00
CCR	0.03	0.06	0.6665	0.00
Control	0.00	.	.	.
**Dependent Variable: T2_Butyrate, *n* = 131**				
**Predictors**	**β**	**SE**	***p*-Value**	R^2
Intercept	0.24	0.22	0.2944	.
T0_Butyrate	0.78	0.09	0.0001	0.39
Age, y	0.00	0.00	0.6386	0.00
sex 1	−0.11	0.06	0.0829	0.01
sex 2	0.00	.	.	.
Q1	0.09	0.09	0.3039	0.01
Q2	−0.04	0.09	0.7005	0.00
Q3	0.00	0.09	0.9628	0.00
Q4	0.00	.	.	.
ICR	0.11	0.08	0.1438	0.01
CCR	0.05	0.08	0.4978	0.00
Control	0.00	0.22	.	.
**Dependent Variable: T3_Butyrate, *n* = 130**				
**Predictors**	**β**	**SE**	***p*-Value**	R^2
Intercept	0.18	0.20	0.3777	.
T0_Butyrate	0.50	0.08	0.0001	0.24
Age, y	0.00	0.00	0.2714	0.01
sex 1	−0.01	0.05	0.9138	0.00
sex 2	0.00	.	.	.
Q1	0.12	0.08	0.1543	0.01
Q2	0.11	0.08	0.1654	0.01
Q3	0.00	0.08	0.9767	0.00
Q4	0.00	.	.	.
ICR	0.04	0.07	0.5780	0.00
CCR	0.04	0.07	0.5927	0.00
Control	0.00	.	.	.

[1] T0_Butyrate, T1_ Butyrate, T2_ Butyrate, and T3_ Butyrate–butyrate concentrations at baseline, wk 12, wk 24 and wk 50 respectively. Q1, Q2, Q3 and Q4–weight loss quartiles 1, 2, 3, and 4 respectively; ICR, CCR

and Control–intermittent calorie restriction, continuous calorie restriction, and control groups respectively of the initial study arms; sex 1-male, sex 2-female. R^2–squared semi-partial correlation coefficient.

Supplemental Table S6. Reported Energy and Macronutrient Intake at Baseline, and Reported Relative Changes Across Quartiles over Time at Week 12 and 50 [1].

		Baseline	Log_e Relative Change [2] (wk 12)	Log_e Relative Change [2] (wk 50)
Energy [kcal/d]	Q1	1834 ± 527.7	−15.5 ± 5.0	−15.4 ± 8.4
	Q2	2069.7 ± 572.2	−22.5 ± 4.3	−23.1 ± 4.6
	Q3	2185.4 ± 621.8	−27.6 ± 3.4	−12.2 ± 10.8
	Q4	2016.1 ± 563.2	−37.8 ± 5.2	−17.4 ± 6.9
Fat [g/d]	Q1	70.9 ± 22.9	−24.5 ± 6.7	−8.5 ± 11.5
	Q2	81.6 ± 26.2	−35.0 ± 6.3	−25.6 ± 6.5
	Q3	90.8 ± 30.8	−47.1 ± 5.1	−21.5 ± 10.3
	Q4	83.7 ± 31.3	−59.4 ± 6.3	−37.3 ± 9.0
Protein [g/d]	Q1	69.4 ± 27.7	−4.0 ± 4.3	3.9 ± 8.8
	Q2	78.1 ± 24.7	−11.3 ± 5.0	−15.2 ± 5.6
	Q3	79.6 ± 17.9	−17.3 ± 3.9	−3.7 ± 9.5
	Q4	75.5 ± 23.6	−24.4 ± 6.0	−11.1 ± 7.8
Carbohydrate [g/d]	Q1	199 ± 63.7	−14.4 ± 7.0	−16.8 ± 8.5
	Q2	223 ± 64.8	−19.7 ± 4.7	-15.6 ± 4.9
	Q3	227.2 ± 78.7	−17.4 ± 4.8	-2.3 ± 13.2
	Q4	212.2 ± 62.9	−26.9 ± 5.5	-2.6 ± 6.7
Fiber [g/d]	Q1	15.5 ± 6.2	12.9 ± 7.0	5.7 ± 9.5
	Q2	19 ± 6.3	11.9 ± 5.4	10.2 ± 7.3
	Q3	17.9 ± 7	19.2 ± 6.9	22.5 ± 12.9
	Q4	18 ± 7.5	19.7 ± 7.4	27.8 ± 10.4
Alcohol [g/d]	Q1	11.2 ± 14.5	−32.1 ± 34.0	−54.5 ± 37.1
	Q2	11.1 ± 14	−15.5 ± 19.9	−73.8 ± 29.7
	Q3	13 ± 13.5	−96.2 ± 37.1	−90.9 ± 39.1
	Q4	8.8 ± 10.1	−160.7 ± 36.9	−73.0 ± 60.3

[1] *n* = 144. Q1-quartile 1; Q2-quartile 2; Q3-quartile 3; Q4-quartile 4. Baseline values are means ± SD. [2] Means (±SEM) of individual loge relative changes between baseline and post-intervention.

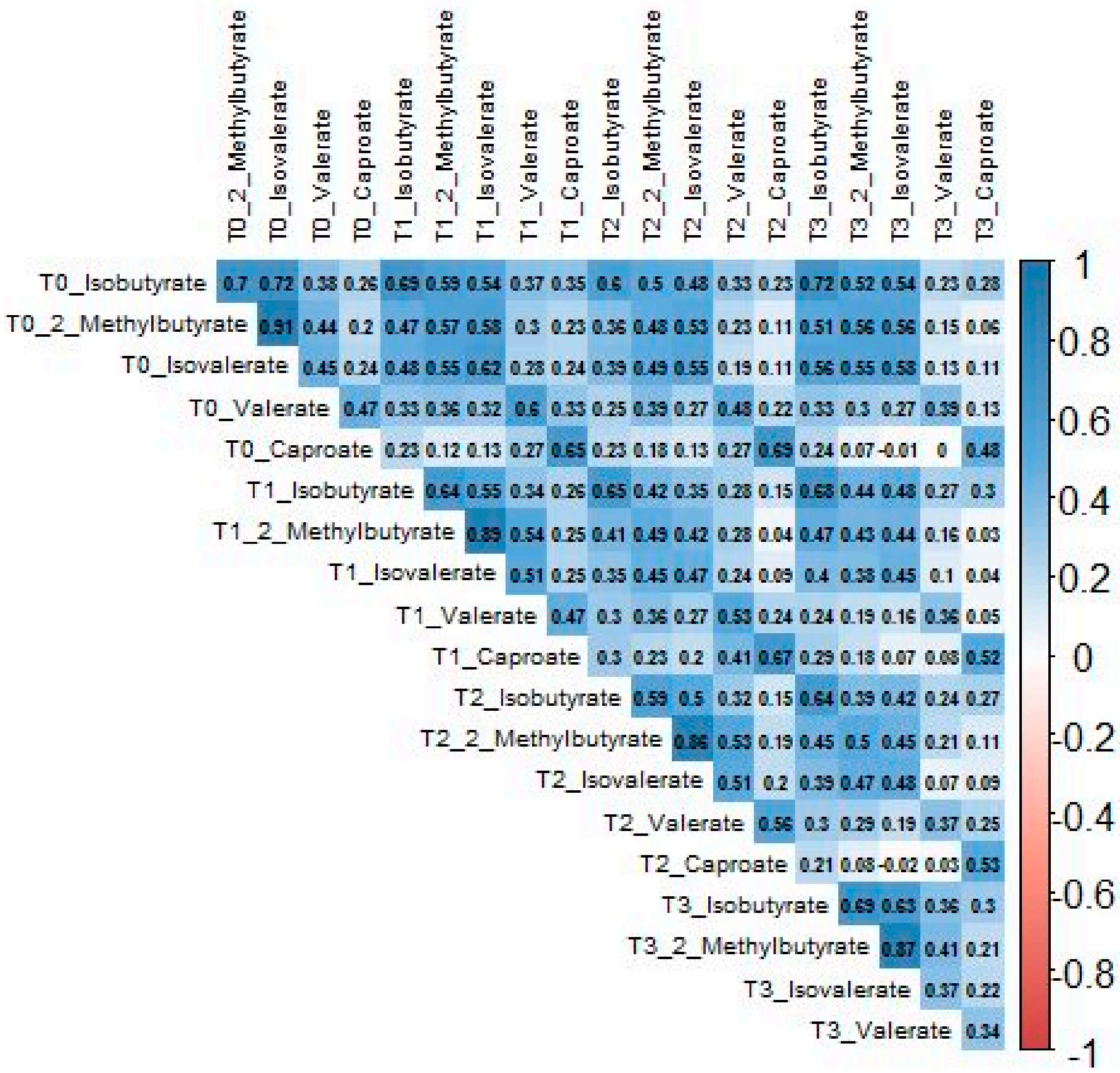

Supplemental Figure S1. Correlations between other SCFAs across time points. T0-baseline, T1-week 12, T2-week 24, T3-week 50.

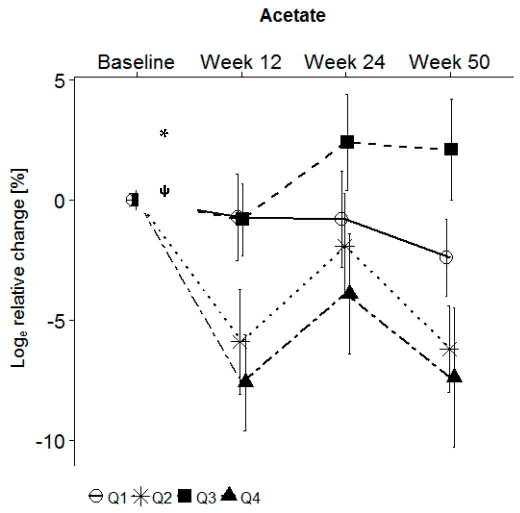

(a)

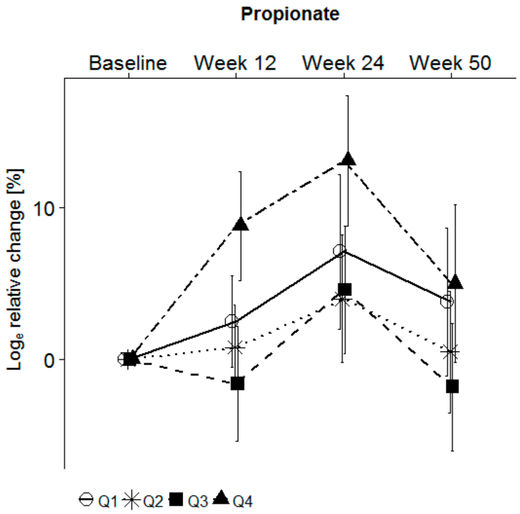

(b)

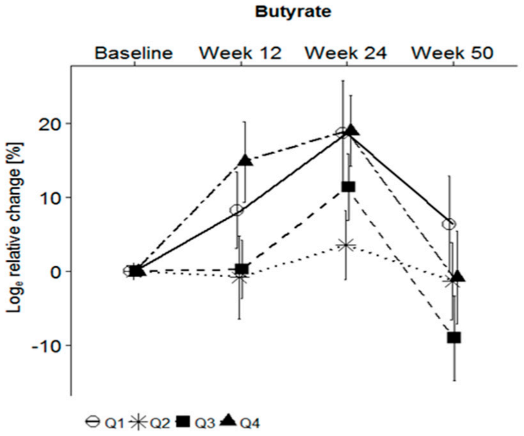

(c)

Supplemental Figure S2. (**a**) Changes in acetate according to weight loss quartiles; (**b**) Changes in propionate according to weight loss quartiles; (**c**) Changes in butyrate according to weight loss. Data are shown as means±SEM of loge percentage changes. Q1, Q2, Q3, Q4 are quartile 1, 2, 3 and 4 respectively. * Significant difference across all quartiles at week 12; φ Significant difference between Q1 and Q4 at week 12.
